# Prevalence of Transfusion Transmitted Virus Infection in Hemodialysis Patients and Injection Drug Users Compared to Healthy Blood Donors in Isfahan, Iran

**DOI:** 10.1155/2012/671927

**Published:** 2012-11-19

**Authors:** Behrooz Ataei, Alireza Emami Naeini, Farzin Khorvash, Mohammad Reza Yazdani, Abbas-Ali Javadi

**Affiliations:** Infectious Diseases and Tropical Medicine Research Center, Isfahan University of Medical Sciences, Isfahan, Iran

## Abstract

*Introduction*. The pathogenicity and transmission routes of Transfusion Transmitted Virus (TTV) remain unclear. The aim of this study was to determine the prevalence of TTV in hemodialysis patients, injecting drug users (IDUs), and healthy blood donors, in Isfahan, Iran. *Method*. In a case-control study, a total of 108 subjects were put into three groups namely Group I, 36 hemodialysis patients; Group II, 36 IDUs; and Group III, 36 healthy blood donors as the control group. A 5 ml blood sample was collected from each subject in an EDTA-containing tube. Samples were tested for TTV DNA by means of real-time polymerase chain reaction (PCR). *Results*. The mean age was 38.7 ± 14.7 years. Seventy-one subjects (66%) were male. Of the108 cases, 30 (27.8%) were TTV positive and 78 (72.2%) were TTV negative. The prevalence of TTV in IDUs [21 (58%)] was significantly higher than in the other groups [group I: 6 (17 %) and group III: 3 (8%)] (*P* < 0.0001). *Conclusion*. The prevalence of TTV in IDUs is significantly higher than in both hemodialysis patients and general population in Isfahan, Iran. It seems necessary to take serious measures to reduce the risk of TTV transmission to IDUs' close contacts and health care providers.

## 1. Introduction 

 Transfusion transmitted virus (TTV) was identified in 1997 in Japan in a patient with acute posttransfusion non-A-to-G hepatitis [1]. 

 TTV is a nonenveloped, single-stranded virus with a wide genetic variety and a worldwide distribution. Its frequency varies in different parts of the world [2]. TTV is believed to be hepatotropic since its viral levels are observed to be higher in the liver than in the serum of infected patients. TTV has also been identified within hepatocytes, and was shown to replicate, by in situ hybridization and PCR; however, only minor morphologic changes have been seen in cells with positive hybridization signals [3]. TTV has been found in many body fluids including saliva, milk, tears and feces [4]. Parenteral [5], fecal-oral [6], mother to fetus and sexual [7] routes have been suggested for TTV transmission.

 It has been shown that TTV infection is associated with increased serum transaminases [8]. Relationship between TTV infection and posttransfusion hepatitis, acute hepatitis, and chronic liver disease with unknown etiology has also been proposed [8, 9]. However, this relationship has not yet been proved [10, 11]. 

 The idea that TTV infection may increase the risk of hepatocellular carcinoma in patients with chronic HCV infection remains to be confirmed [12]. On the other hand, high prevalence of TTV infection in control groups of several studies makes its pathogenic role more questionable [2]. Therefore, more studies, for example, cohort, experimental, and so forth, are needed to determine whether or not TTV infection is associated with liver disease.

 Although TTV can be transmitted through blood or blood products, these routes of transmission do not explain the high prevalence of TTV in blood recipients in some parts of the world such as Japan (12%) and Scotland (1.9%) [4, 13]. Parenteral transmission for TTV has not yet been completely accepted and therefore more studies are required to gain certainty in this regard.

 As the first step in all studies on the pathogenesis and transmission of TTV, it is necessary to evaluate the prevalence of TTV among high risk groups and healthy individuals in the community. 

 Patients on maintenance hemodialysis and intravenous drug users (IDUs) are particularly predisposed to blood-borne infections and therefore are suitable populations for clinical and epidemiologic studies on the newly discovered TTV.

 The prevalence of TTV in different countries ranged from 2% to 53% [14–18] in hemodialysis patients and 19% to 43% in IDUs [19–21].

 Geographical distribution, diagnostic methods, size of study groups, and the presence of clinical and demographic aspects are among the causes of this wide range. In early studies, the prevalence of TTV was reported 1% to 40% in healthy blood donors [4]. As more inclusive primers were used to detect differing genotypes, the reported prevalence among blood donors increased dramatically, approaching 100% in some studies [22]. 

 Unfortunately there is no report on the prevalence of TTV in hemodialysis patients, IDUs, and healthy people in Isfahan, central Iran, yet. 

 Moreover, with respect to lack of comprehensive information on the pathogenesis of TTV and different transmission routes (parenteral, fecal oral, and sexual) for TTV, a probably high prevalence of TTV in the aforementioned groups requires serious measures to be taken in order to minimize the risk of transmission to their families, their close contacts, and health care providers. 

## 2. Material and Method

 This unmatched case-control study was carried out at Al-Zahra and Noor hospitals, Isfahan, Iran, in 2010. The purpose of this study was to compare the prevalence of TTV in hemodialysis patients, IDUs, and healthy blood donors. A total of 108 cases were categorized into three groups: Group I; 36 hemodialysis patients (randomly selected from hemodialysis units at Al-Zahra and Noor hospitals, Isfahan, Iran), Group II; 36 IDUs (randomly selected from addicts under surveillance by the Infectious Diseases and Tropical Medicine Research Center, Isfahan, Iran, and Group III; 36 healthy blood donors (randomly selected from people who had referred to Isfahan's blood bank) as a control group. IDUs with history of thalassemia, hemophilia, or being frequent recipients of blood and blood products and those under treatment for hepatitis B or C were excluded from the study. Also subjects in Group III who had a history of acute or chronic diseases were excluded. 

 Medical Ethics Committee approved our trial and every individual in the study was asked to sign a letter of consent.

 Demographic data were collected through face to face interview. A 5 mL blood sample was collected from each subject on an EDTA-containing tube and was transferred to the laboratory. Then DNA extraction was performed using DNA extraction kit, High Pure Viral Nucleic Acid Kit (Roche Diagnostics, Mannheim, Germany). Subsequently, DNA reproduction was performed using specific primers and probe by Rotor-Gene 6000 real-time PCR instrument (Corbett Research, Sydney, Australia).

 Statistical analysis was done on a computer using SPSS16. Subjects were not matched for age, sex, and other demographic variables among groups. All data are presented as mean ± SD and number (%) as required. One way ANOVA was used to compare age between groups. Also sex and TTV percentage were assessed via Pearson chi-square. Statistical significance was accepted at *P* < 0.05, two tailed. 

## 3. Results

 All 108 subjects of the three groups were included in our analysis. The mean age was 38.7 ± 14.7. Seventy-one (66%) were male and 37 (34%) were female.  Table 1 shows characteristics of subjects among the study groups, mean age, and frequency of male and female among groups were statistically significant.

 Among all, 30 (27.8%) were TTV positive and 78 (72.2%) were TTV negative.

 TTV positivity in the study groups are shown in Table 2. Results were compared between groups using the chi-square test. The number of TTV positive subjects in Group II was more than in other groups with a statistically significant difference (*P* < 0.001). Difference in the frequency of TTV between Group I and Group III was not statistically significant (*P* = 0.47). 

## 4. Discussion

 In this study, prevalence of TTV in hemodialysis patients, IDUs, and blood donors in Isfahan, Iran, was surveyed. The results showed that prevalence of TTV in IDUs was higher than in the other groups. Significant differences between “IDUs versus blood donors” and “IDUs versus hemodialysis patients” were found. However the difference between hemodialysis patients and blood donors was not statistically significant. 

Studies on the epidemiology of TTV in hemodialysis patients and IDUs have resulted in different findings. Differences in the spread pattern of infection in various geographical regions, different inclusion criteria, transmission routes of TTV, and difference in primers used for diagnosis can explain the variable results.

 Prevalence of TTV among hemodialysis patients ranged from 2.5% to 53% in various studies [14–18]: 2.5% in the study by Halfon et al. in France [14], 53% in the study by Forns et al. in Spain [15], 30% and 51.3% in studies by Oguchi and Utsunomiy in Japan [16, 17], and 9.3% in the study by Kheradpezhouh et al. in Tabriz, north-western Iran [18]. In our study, the prevalence of TTV in hemodialysis patients was 16.7%. 

 Prevalence of TTV in IDUs was reported to be 19–43% in various studies [19–21]: 40% in the study by Naoumov et al. in England [19], 19.2% in the study by Biagini et al. in France [20], and 43.3% in the study by Soudbakhsh et al. in Tehran, capital of Iran [21]. In our study, the prevalence of TTV in IDUs was 58.3%.

 Prevalence of TTV in healthy blood donors ranged 1–40% in early studies [4]. As more inclusive primers were used to detect differing genotypes, the reported prevalence among blood donors increased dramatically, approaching 100% in some studies [22]. Some reported prevalence of TTV in the healthy blood donors is as follows: 94% in the study by Vasilyev et al. in Russia [23], 41% in the study by Zandie et al. in Tehran, capital of Iran [24], and 23.7% in the study by Jalali Far et al. in Ahwaz, south-western Iran [25]. In our study, the prevalence of TTV in healthy blood donors was 8.3%. The fact that TTV is also detectable in healthy population confirms the role of nonparenteral routes of transmission.

 In this study, prevalence of TTV in IDUs was significantly higher than in healthy blood donors. This strongly suggests that parenteral route, as in injection drug use, can play an important role in TTV transmission. 

 According to this study, prevalence of TTV in IDUs is significantly higher than in hemodialysis patients and also in general population in Isfahan, Iran. Clinical significance of TTV cannot be evaluated based on this study; however it could serve as a basis for future studies (e.g., cohort and experimental) on the pathogenesis of TTV. In the absence of comprehensive information on the pathogenesis of TTV along with the variety of transmission routes (parenteral, fecal oral, and sexual), and the high prevalence of TTV in IDUs, it seems necessary to develop measures to minimize the risk of TTV transmission to IDUs' families, their close contacts, and health care providers. 

## Figures and Tables

**Table 1:** Characteristics of study groups.

	Group I (*n* = 36)	Group II (*n* = 36)	Group III (*n* = 36)	*P* value
Mean Age	50.5 ± 15.4	35.2 ± 11.1	30.33 ± 8.5	<0.0001
Sex				
Male	26 (72)	33 (92)	12 (33)	< 0.0001
Female	10 (28)	3 (8)	24 (67)	

Data presented as means ± 1SD and number (%). Group I: hemodialysis patients; Group II: intravenous drug user diseases; Group III; healthy blood donors.

*P* values calculated with one way ANOVA and chi-square test.

**Table 2:** Comparison of TTV positivity among the study groups.

	TTV positive	TTV negative	*P* value
Group I (*n* = 36)	6 (17)	30 (83)	
Group II (*n* = 36)	21 (58)	15 (42)	<0.0001
Group III (*n* = 36)	3 (8)	33 (92)	

Data presented number (%). Group I: hemodialysis patients; Group II: intravenous drug user diseases; Group III; healthy blood donors.

*P* values calculated with chi-square test. Statistical significance was observed between Groups 1 and 2 (*P* value < 0.0001) and Groups 2 and 3 (*P* value < 0.0001). Difference between Groups 1 and 2 was not statistically significant (*P* value = 0.47).
